# Developing a Screening Procedure During the COVID-19 Pandemic: Process and Challenges Faced by a Low-Incidence Area

**DOI:** 10.3389/fmed.2021.654754

**Published:** 2021-12-24

**Authors:** Wei Tang, Fei Wang, Jian-Wei Wang, Yao Huang, Li Liu, Shi-Jun Zhao, Xin-Ming Zhao, Ning Wu

**Affiliations:** ^1^Department of Diagnostic Radiology, National Cancer Center, National Clinical Research Center for Cancer, Cancer Hospital, Chinese Academy of Medical Sciences and Peking Union Medical College, Beijing, China; ^2^Office of Cancer Screening, National Cancer Center, National Clinical Research Center for Cancer, Cancer Hospital, Chinese Academy of Medical Sciences and Peking Union Medical College, Beijing, China; ^3^Department of PET-CT Center, National Cancer Center, National Clinical Research Center for Cancer, Cancer Hospital, Chinese Academy of Medical Sciences and Peking Union Medcial College, Beijing, China

**Keywords:** COVID-19, screening, computerized tomography (CT), healthcare [MeSH], hospital (clinic)-clinic corporation

## Abstract

**Purpose:** To summarize the imaging results of COVID-19 pneumonia and develop a computerized tomography (CT) screening procedure for patients at our institution with malignant tumors.

**Methods:** Following epidemiological investigation, 1,429 patients preparing to undergo anti-tumor-treatment underwent CT scans between February 17 and April 16, 2020. When CT findings showed suspected COVID-19 pneumonia after the supervisor radiologist and the thoracic experience radiologist had double-read the initial CT images, radiologists would report the result to our hospital infection control staff. Further necessary examinations, including the RT-PCR test, in the assigned hospital was strongly recommended for patients with positive CT results. The CT examination room would perform sterilization for 30 min to 1 h. If the negative results of any suspected COVID-19 pneumonia CT findings were identified, the radiologists would upload the results to our Hospital Information Systems and inform clinicians within 2 h.

**Results:** Fifty (0.35%, 50/1,429) suspected pneumonia cases, including 29 males and 21 females (median age: 59.5 years old; age range 27–79 years), were identified. A total of 34.0% (17/50) of the patients had a history of lung cancer and 54.0 (27/50) underwent chemotherapy or targeted therapy. Forty-six patients (92.0%) had prior CT scans, and 35 patients (76.1%) with suspected pneumonia were newly seen (median interval time: 62 days). Sub-pleura small patchy or strip-like lesions most likely due to fibrosis or hypostatic pneumonia and cluster of nodular lesions were the two main signs of suspected cases on CT images (34, 68.0%). Twenty-seven patients (54.0%) had, at least once, follow-up CT scan (median interval time: 18.0 days). Only one patient had an increase in size (interval time: 8 days), the immediately RT-PCR test result was negative.

**Conclusion:** CT may be useful as a screening tool for COVID-19 based on imaging features. But the differential diagnosis between COVID-19 and other pulmonary infection and/or non-infectious disease is very difficult due to its overlapping imaging features.The confirmed diagnosis of the COVID-19 infection should be based on the etiologic eventually. The cancer patients at a low-incidence area would continue treatment by screening carefully before admission.

## Introduction

Since December 2019, the Novel Coronavirus (COVID-19) has caused an outbreak of pneumonia and has rapidly spread across the globe. On 11 March 2020, the WHO, declared COVID-19 to be a pandemic (1). Globally, as of 2:10 p.m. CET, 13 March 2021, there have been 118,754,336 confirmed cases of COVID-19, including 2,634,370 deaths, reported to WHO. While the number of confirmed and suspected cases continues to grow, the rate of increase is now gradually declining in some areas. On March 19, China reported no new domestic cases for the first time, and the first prevailing peak period of the pandemic was over. Thanks to the efforts of the Chinese government, numerous measures such as preventing the spread and import of the infection, strict restrictions on the gathering of large numbers of people, disinfecting public areas, and taking charge of symptomatic people (such as those with fever and/or cough) has led to Beijing, the capital of China, maintaining its status up until mid-April, 2020 as a low-incidence area of COVID-19 infections during the period of outbreak in China.

The epidemiologic, laboratory, and clinical features of COVID-19 pneumonia have been described (2–5). Because a curative vaccine has not yet been developed, early detection and efficient control of the route of transmission (i.e., isolation of suspected cases, and disinfection) remain the most effective ways to fight the outbreak. The WHO recommends suspected cases be screened for the virus with nucleic acid amplification tests (NAAT) (6), such as reverse-transcriptase polymerase chain reaction (RT-PCR). Laboratory detection is time-consuming and may not be available for all those with a suspected infection owing to the shortage of test kits for SARS-CoV-2. These challenges increase the risk of spread by free movement of people highly suspected of carrying the disease. In addition, the laboratory test can give false negative results. CT is considered the first-line imaging modality in highly suspected cases and is helpful in monitoring imaging changes during treatment. Therefore, CT has been identified as an efficient clinical diagnostic tool for people with suspected COVID-19 (7).

Sensitivity and specificity of chest CT for COVID-19 are reported to range from 80 to 90% and 60 to 70%, respectively (8, 9). The diagnosis and treatment program (6th version), published by the National Health Commission of the People's Republic of China (10), defined the diagnosis of viral pneumonia based on radiologic features by radiologists as one of the diagnostic criteria for COVID-19.

The Cancer Hospital Chinese Academy of Medical Sciences (CHCAMS) & National Cancer Center of China (NCC) is one of the leading specialist cancer hospitals of China. NCC has over 1,000,000-patient output annually. In 2019, 79,398 X-ray, 210,503 CT, and 49,988 MRI examinations were performed in the center. Patients with cancer have an increased risk of complication and death during or after treatment related to COVID-19 infection (11, 12). During the outbreak period, a large proportion of the examinations, diagnosis, treatments and curative effect evaluations of patients were delayed. It is very important for the patients with malignant tumor that the prevention and control of COVID-19 infection, especially when they undergo therapies such as chemotherapy, radiotherapy, targeted therapy or immunotherapy. Our hospital has made any efforts to minimize any possibilities of in-hospital transmission to ensure a safe environment for both patients and staff. In order to make the patients with malignant tumor get the necessary treatment on time, the infection control committee of NCC/CHCAMS decided to use CT to screen the COVID-19 pneumonia for pre-treatment patients as a necessary examination on February 16, 2019.

To our knowledge, few articles which focuses on the COVID-19 CT screening for cancer patients had been published (13). Now, we will want to share our experience and understanding with medical peers, which will contribute directly to the accumulation of experience in other medical facilities.

## Materials and Methods Study

### Design and Patients

The questionnaire survey which designed by our hospital infection control team were written by each pre-anti-tumor treatment patients. This questionnaire included personal information (gender, age, telephone number, home or permanent address, exact date of arrival Beijing, and duration from arrival in Beijing to the current date) and epidemiological investigation. The epidemiological investigation included (10): (i) whether the patient had been to Hubei Province within the past 2 weeks; (ii) whether the patient had taken public transport which passed through Hubei Province within the past 2 weeks; (iii) whether the patient had had contact with anybody from Hubei Province within the past 2 weeks; (iv) whether the patient had been abroad or had had contact with anybody who coming overseas; (v) whether the patient had had contact with COVID-19 confirmed or suspected cases; (vi) appearance or worsening of symptoms such as fever and body temperature; (vii) whether the patient had taken any antipyretics during the fever; and (viii) whether the patient displayed typical symptoms such as fever, sore throat, nasal congestion, malaise, headache, myalgia, or dyspnea. Given that our hospital was not a designated hospital for COVID-19 infection patients, if patients or those accompanying them gave any positive responses, they would be declined entry. If the patients or those accompanying them had a fever higher than 37.3°C, and had no alternative explanation for these symptoms, COVID-19 testing via a SARS-CoV-2 quantitative PCR nasopharyngeal swab was sent in addition to a respiratory pathogen panel PCR at the nearby fever clinic.

Finally, in this center, a total of 1,429 patients with cancer history underwent the CT procedure and routine blood test between February 17 and April 16, 2020. All patients denied any direct exposure history to Wuhan or to people who had a direct exposure history to Wuhan (i.e., long-term exposure history to Wuhan, or travel to Wuhan before diagnosis) and no one showed any symptoms associated with COVID-19.

### Imaging Technique

All patients underwent scanning with the following four scanners: Optima 660, LightSpeed VCT, Discovery 750 HD (GE Healthcare, Milwaukie, USA), and Somatom Edge (Siemens Healthcare, Erlangen, Germany). Instead, the routine CT scan of Low- dose CT scan was used. The acquisition parameters were set at 120 kVp; 150-250 mAs; pitch, 0.938–1.0; and collimation, 0.625 mm. All imaging data were reconstructed by use of a standard reconstruction algorithm with a slice thickness of 0.625–1.0 mm. All patients were scanned in the supine position wild holding their breath at the end of inspiration. The field of view was set from the apex to the base of the lungs. Images were sent to the Picture Archiving and Communication System (PACS) system to be read by the radiologists.

### Computed Tomography Scan Procedure and Imaging Interpretation

**Figure 1:**
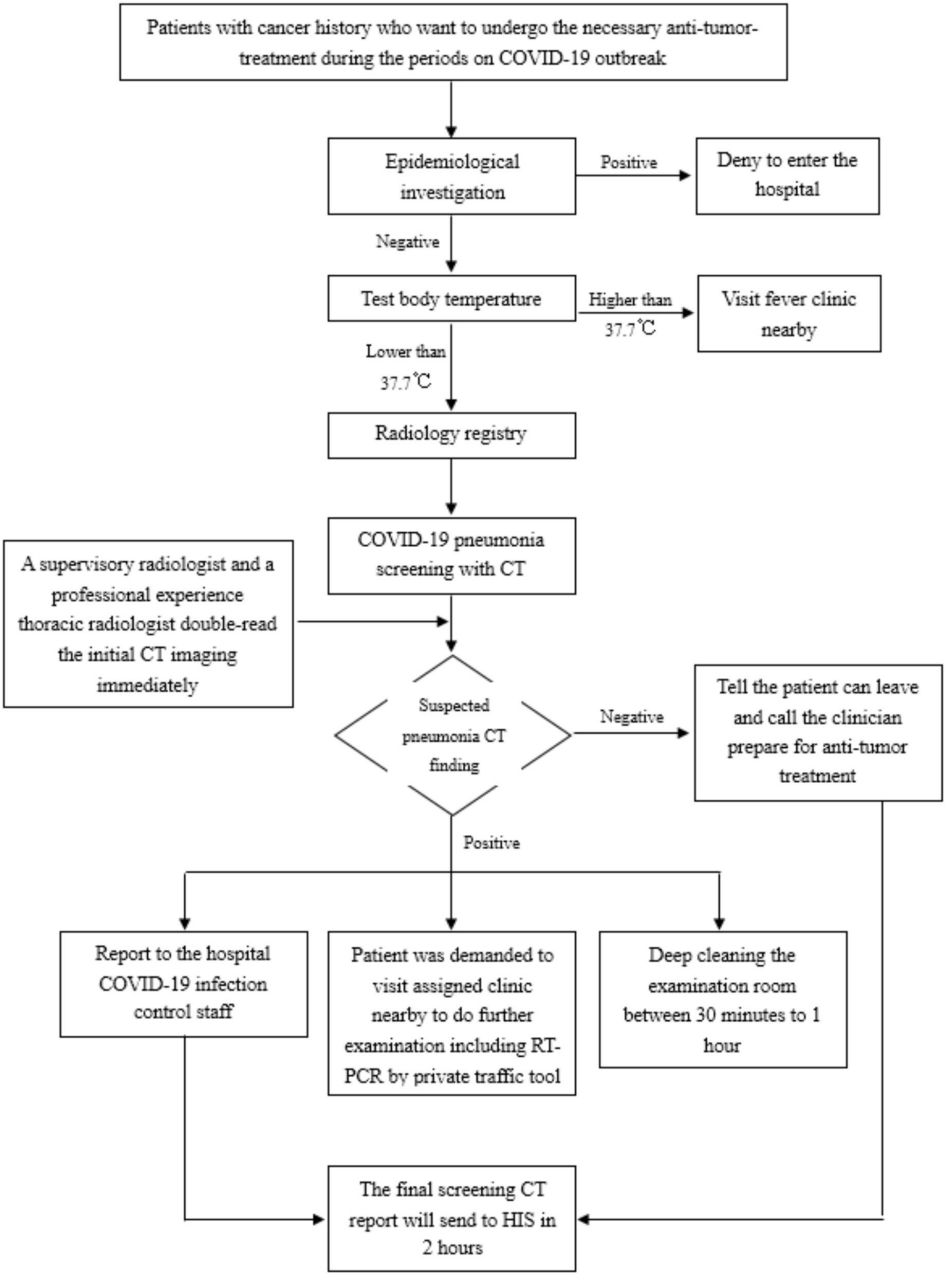
The procedure for COVID-19 screening CT.

To detect suspected COVID-19 on CT imaging, we arranged for several Supervisory Radiologists (SR) to read the CT images immediately after completion of the scan. The SR confirmed whether the CT images showed signs of any type of pneumonia, including bacterial pneumonia, viral pneumonia, and other types, including suspected COVID-19. Comparison was made with prior CT images, in order to confirm whether the lesion was newly seen, persistently existed, or had changed in size or density. All images were viewed with both lung (width, 1,500 HU; level, −700 HU) and mediastinal (width, 350 HU; level, 40 HU) settings. The patient was not permitted to leave the scan room until the technologist of radiology (TR) had obtained permission from the SR. If any CT images showed suspected pneumonia, the SR would ask one of the Professor Radiologists (PR, Y. Huang, JW. Wang, SJ. Zhao, and L. Liu, each of whom have over 10 years of thoracic-imaging diagnosis experience) to immediately read the images again. If the PR confirmed the suspected diagnostic of viral pneumonia, the SR would immediately report the details, including the CT findings and blood test results, to the clinician and the hospital infection control staff. The clinician would then order immediate isolation of the patient for clinical monitoring and treatment. The TR would request the patients attend the nearby fever clinic (given that our center does not have a fever clinic) while avoiding use of public transportation to do the further examination including RT-PCR. The hospital infection control staff would document the information to follow up the results of the patient. Sterilization would be performed: room downtime was typically 30 min to 1 h for room decontamination and passive air exchange. In addition, the final CT report would be double-read by a further two radiologists. The imaging features defined in a previous study (14): ground-glass opacities (GGO), consolidation, mixed GGO and consolidation, centrilobular nodules, architectural distortion, cavitation, tree-in-bud, bronchial wall thickening, reticulation, subpleural bands, traction bronchiectasis, intrathoracic lymph node enlargement, vascular enlargement in the lesion, and pleural effusions (Figure 1 refers to the procedure for COVID-19 screening CT).

### Follow-Up CT Scan

We defined three types of imaging changes: no change, progressive change, and improvement change. No change referred to no obvious changes presented in the chest CT progressive change referred to the presence of new lesions or the presence of extent involvement area during treatment; and improvement change referred to the continually absorbed abnormities. We also evaluated the duration of imaging progress, which was calculated from the time of baseline CT or the time of CT showing new lesions to that of the CT showing abnormal imaging findings.

## Results

### Patients

Among the 1,429 COVID-19 infection screening cases for pre-inpatients, 50 cases (0.35%, 50/1,429) of suspected virus pneumonia in CT imaging, which had been double-read by SR and PR, were reported to the hospital infection control staff. The total of 50 suspected virus pneumonia patients (29 males, 21 females; median 59.5 years old; age range 27–79 years) were included in analysis and the cancer history. Treatment history before CT scan were listed in Table 1. A total of 34.0% (17/50) of the patients had a history of lung cancer, 18.0% (9/50) had a history of breast cancer. Fifty-two percentage (26/50) patients underwent surgical resection, 54.0% (27/50) underwent chemotherapy or targeted therapy, 16.0% (8/50) underwent radiotherapy, and one patient (4.0%) with hepatocellular carcinoma underwent interventional therapy. Thirty-six patients (72.0%) were natives of Beijing and 14 patients (28.0%) were from outside of Beijing, including one patient from Hubei Province. They had complied with 2-week home quarantine requirements before the CT scan. All available clinical, laboratory examinations, and epidemic characteristics were collected.

**Table 1:** Malignant tumor and the treatment history of the patients.

	**Surgical resection (*n* = 26)**	**Chemotherypy/Targettherypy (*n* = 27)**	**Radiotherapy (*n* = 8)**	**Interventinoal therapy (*n* = 1)**
Lung cancer (*n* = 17)	6	12	1	0
Breast cancer (*n* = 9)	8	1	1	0
Esophagus cancer (*n* = 4)	2	2	2	0
Gastric cancer (*n* = 3)	2	1	0	0
Ovarian cancer (*n* = 3)	2	3	0	0
Endometrial cancer (*n* = 2)	1	1	1	0
Hepatocellular carcinoma (*n* = 2)	1	0	0	1
Non-Hodgkin Lymphoma (*n* = 2)	0	2	0	0
Gallbladder carcinoma (*n* = 1)	1	1	0	0
Colon cancer (*n* = 1)	1	0	0	0
Hodgkin lymphoma (*n* = 1)	0	1	0	0
Cervical cancer (*n* = 1)	0	1	1	0
Thymic carcinoma (*n* = 1)	0	1	1	0
Clear cell carcinoma of kidney (*n* = 1)	1	0	0	0
Nasopharyngeal carcinoma (*n* = 1)	0	1	1	0
Olfactory neuroblastoma (*n* = 1)	1	0	0	0

### Initial COVID-19 Screening CT Scan

In our retrospective cohort of 50 patients, 46 patients (92.0%) had prior CT scans. Thirty-five patients (76.1%, 35/46) with suspected pneumonia were newly seen (interval time: 26–145 days, median: 62 days), eight patients (17.4%, 8/46) had slightly increased in size or density (interval time: 13–144 days, median: 74 days), two patients (4.3%, 2/46) had no change, and one (2.2%, 1/46) had increased in size when compared with the recent examination.

**Figure 2:**
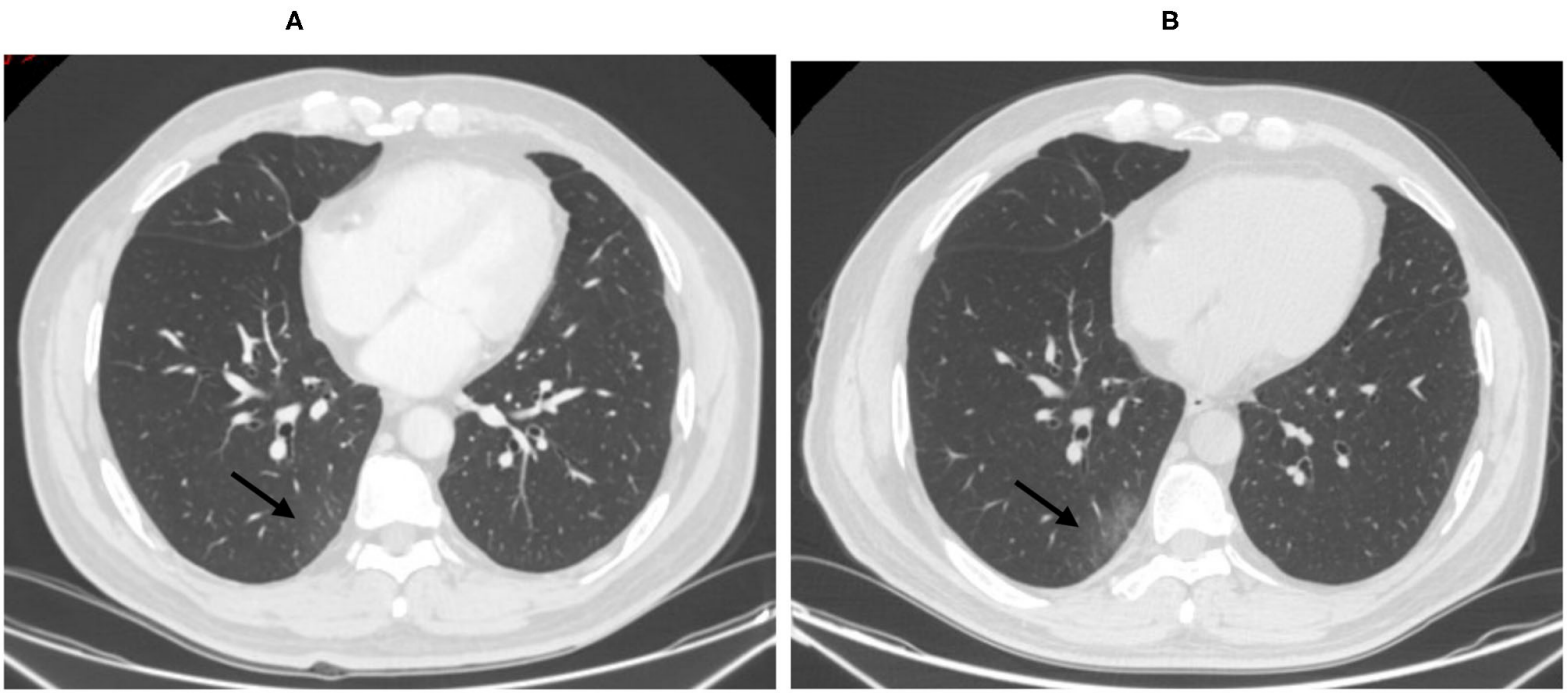
Fifty-six-year-old male with lung adenocarcinoma history. Screening CT images **(B)** showed subpleural bandlike areas of ground glass opacity (GGO) had been increased in size compare to the prior CT scan **(A)** (arrow).

**Figure 3:**
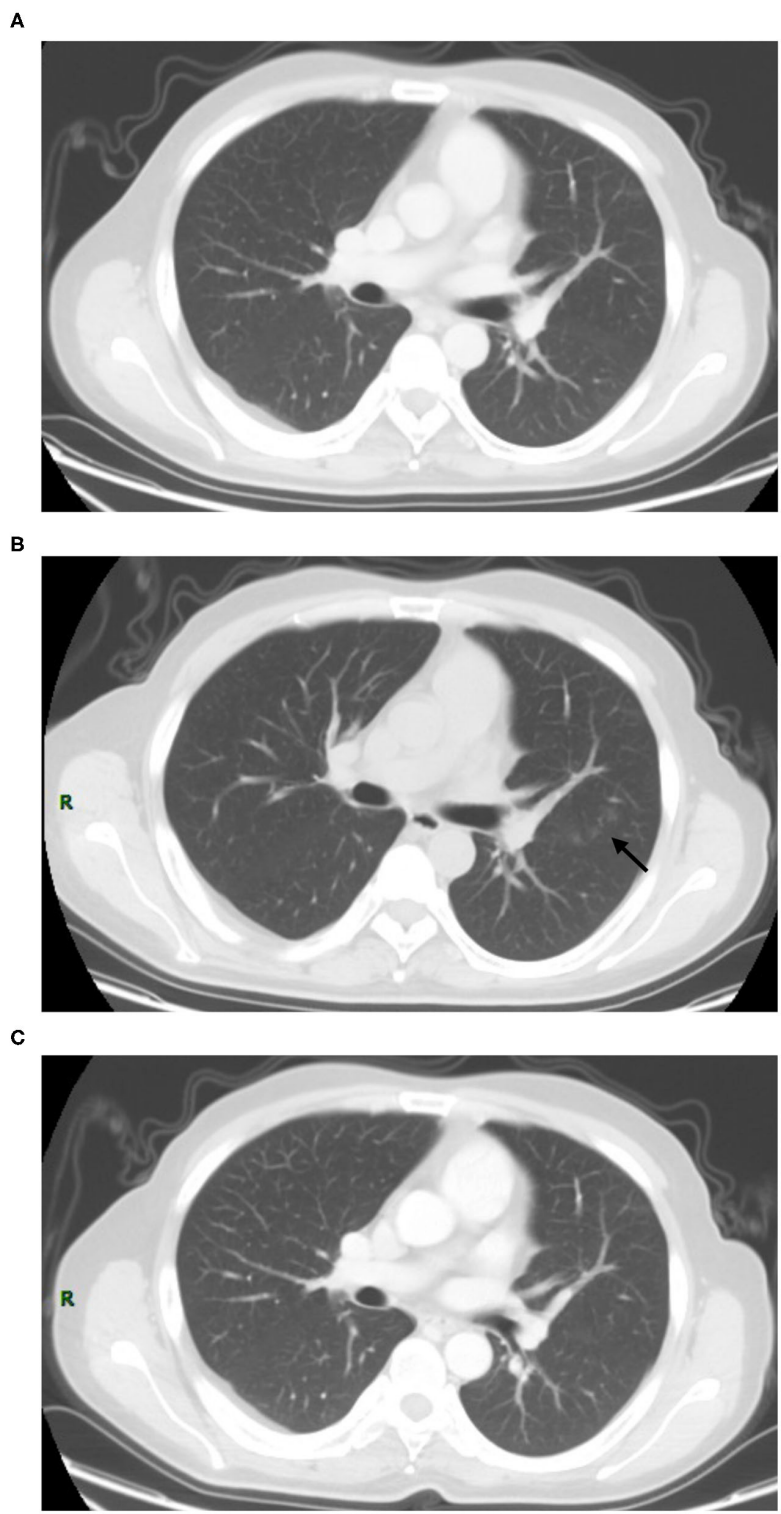
Thirty-seven-year-old male with nasopharynx cancer. Patchy ground glass opacities affecting the left upper lobe had been newly seen **(B)** (arrow) when compared to the prior CT which performed at 2 months ago **(A)**. The short-term CT scan **(C)** showed the lesion had been completely resolved.

**Figure 4:**
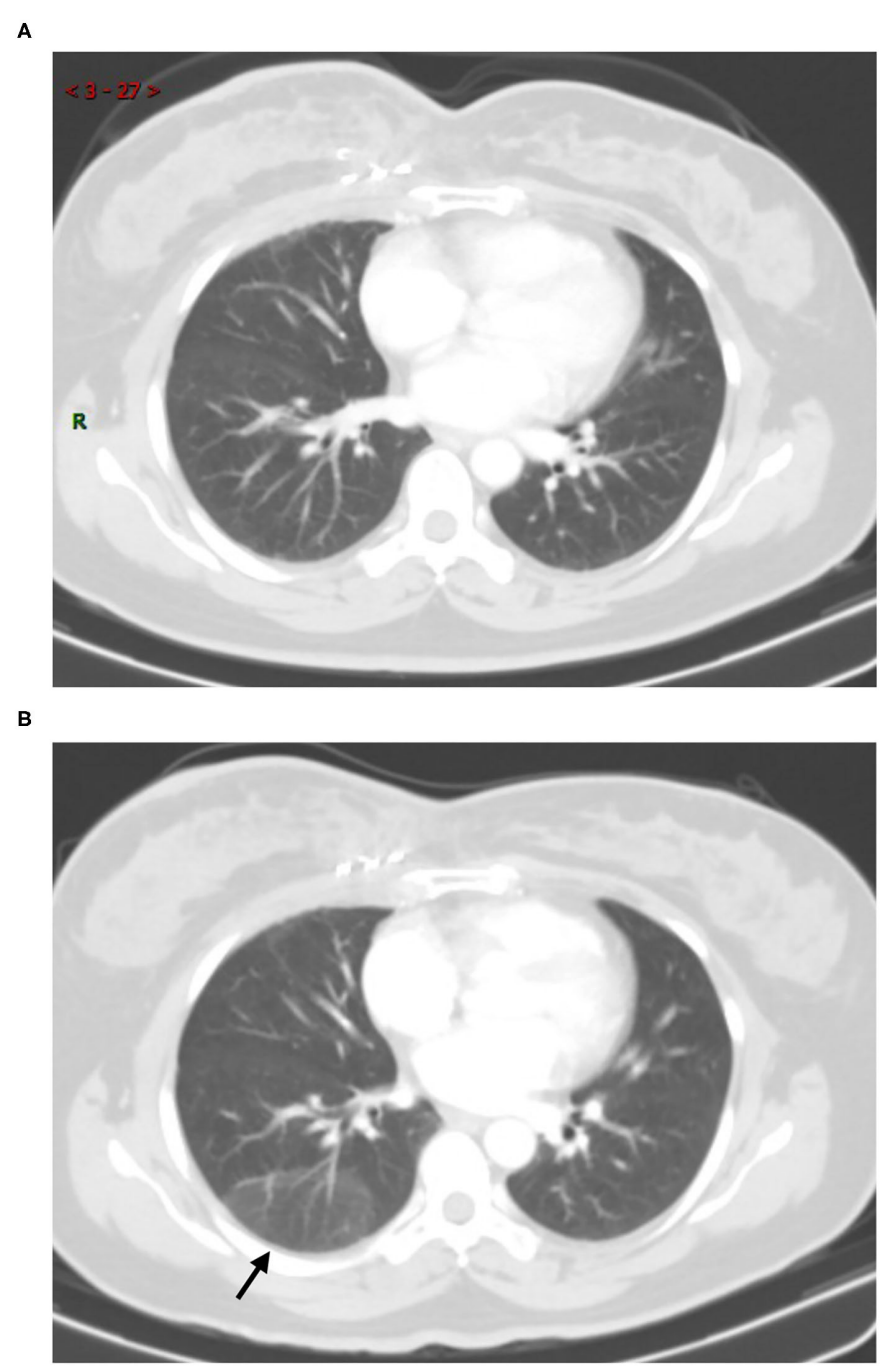
Fourty-two-year-old female with breast cancer history. Screening CT scan showed a focal peripheral rounded and ill-defined GGOs in the right lower lobe [**(B)**, arrow]. Comparison to the prior CT scan **(A)**, it has been newly seen.

**Figure 5:**
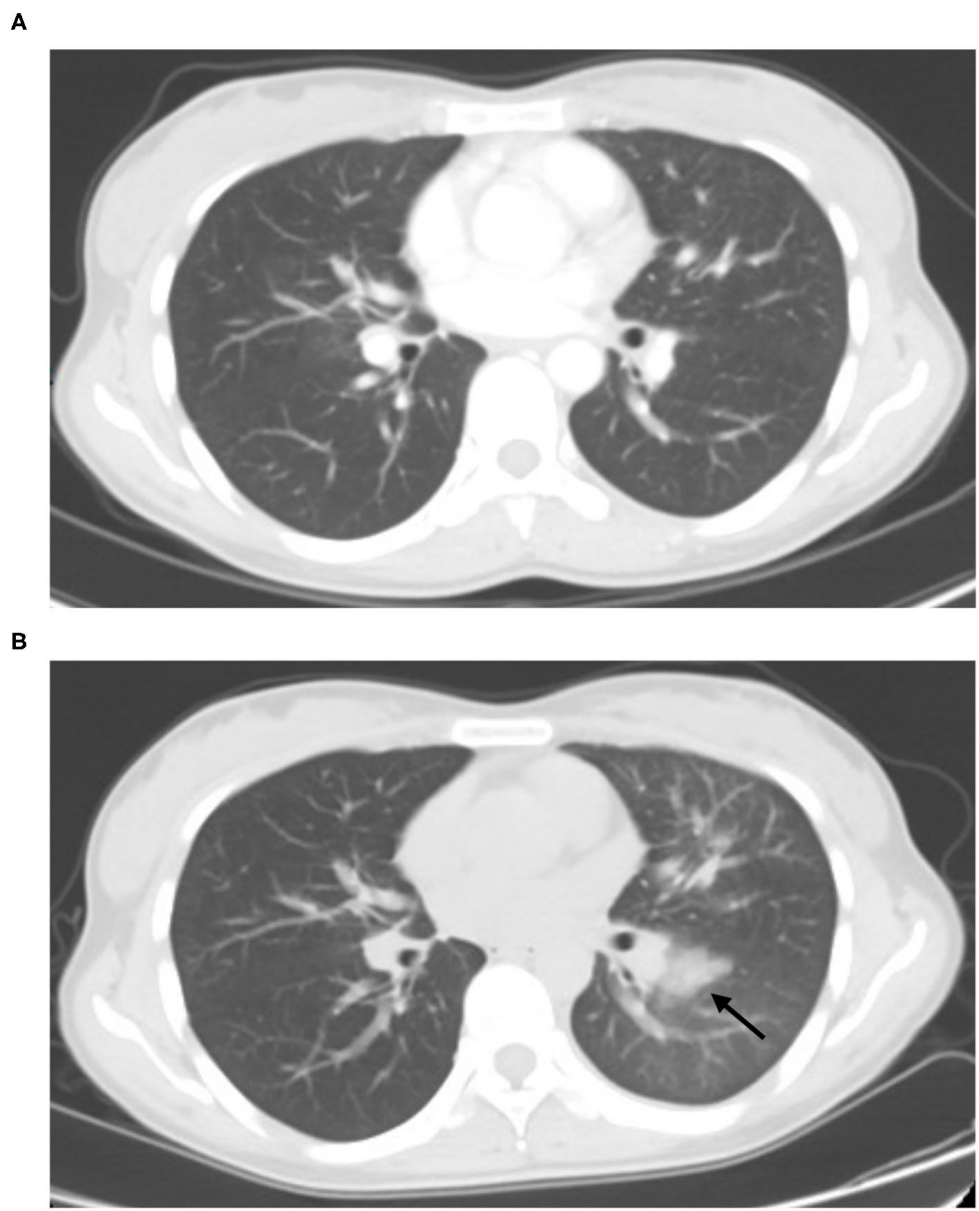
Twenty-seven-year-old female with post-chemotherapy of lung cancer history. Comparison to the prior CT scan **(A)** which performed at 2 months ago, the focal left lower lobe subpleural consolidations was identified [**(B)**, arrow].

**Figure 6:**
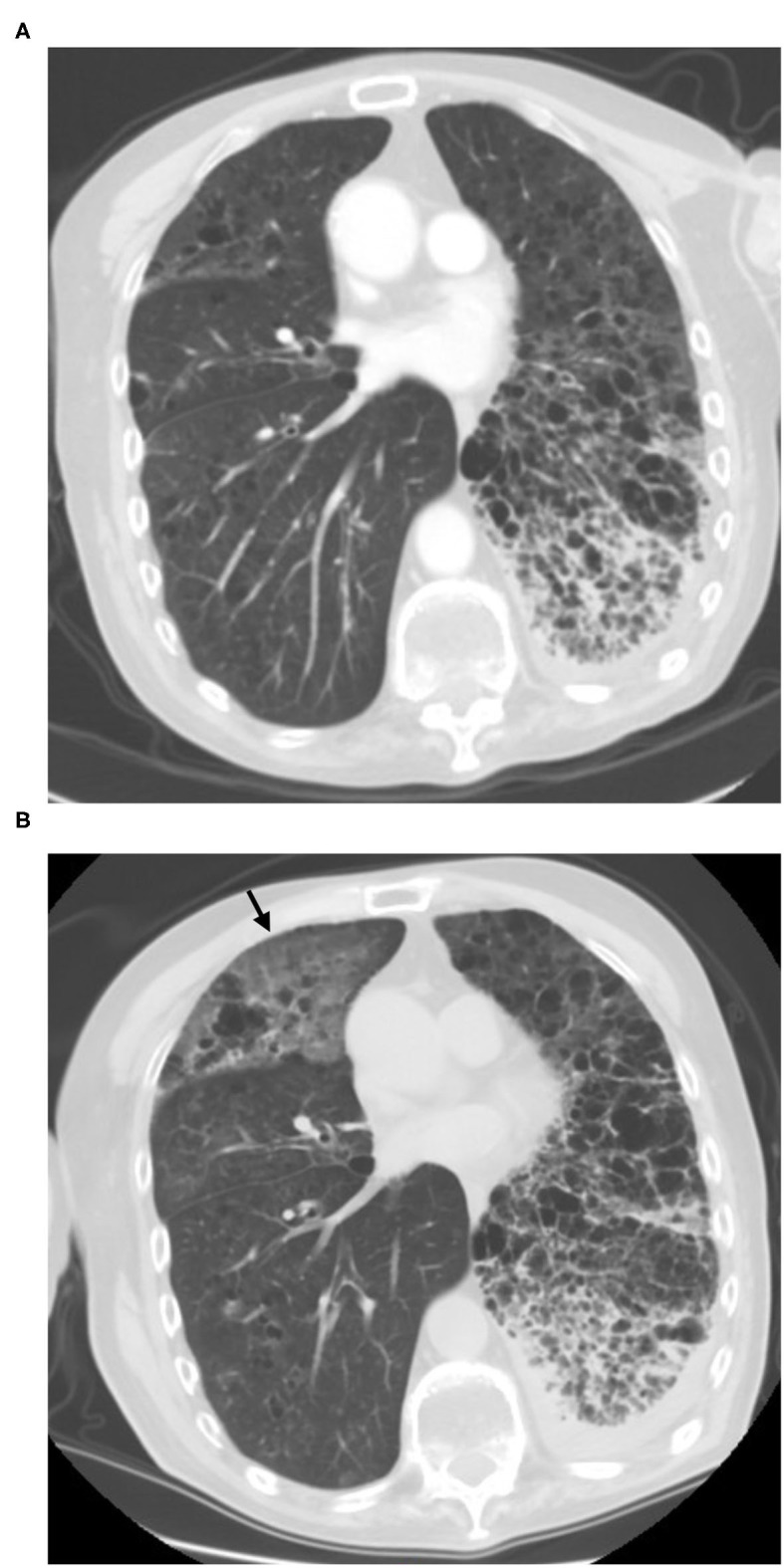
Sixty-six-years-old male with post-target therapy of lung adenocarcinoma history. Comparison with the transverse CT scan **(A)**, the screening CT **(B)** shows new bilateral, peripheral GGO associated with smooth interlobular and intralobular septal thickening (crazy-paving pattern) (arrow).

In COVID-19 screening CT, all lung segments may be involved; there was a slight predilection for the left lower lobe (30 (33.0%) of 91 affected segments among all patients). Involvement of lower lobes (right 48.0 vs. left 60.0%) was more than that of the upper lobes (right 20.0 vs. left 40.0%). Twenty-nine (58.0%) patients had single lobe involvement, 10 (20.0%) patients had involvement of two lobes, and 11 (22.0%) patients had three or more lobes involved. The lesions were predominantly peripheral and subpleural in 29 (58.0%) patients. Sub-pleura small patchy (Figure 2) or strip-like lesions most likely due to fibrosis or hypostatic pneumonia and cluster of nodular lesions (Figure 3) were two main signs of suspected cases on CT images (34, 68.0%). Fourteen cases (28.0%, 14/50) manifested GGO (Figure 4) and consolidation (Figure 5). One case (2.0%, 1/50) manifested interstitial inflammation (Figure 6) and another case (2.0%, 1/50) manifested diffuse small airway lesions most likely due to respiratory bronchiolitis. No patients had mediastinal lymphadenopathy or pleural effusion. Cavitation and tree-in-bud were absent in our cohort. In addition, a part-solid pulmonary nodule with a primary lung cancer imaging feature was identified in a patient with a history of esophagus cancer.

### Follow Up COVID-Screening CT Scan

A total of 85 chest CT examinations on 27 patients had been performed with at least once follow up CT scan as of April 16, 2020. The mean number of CT examinations per patient was 1.7 (85/50). The mean time between the initial and follow up CT studies was 18.0 days (range, 6–53 days). Only one patient had increased in size (interval time: 8 days) compared with the previous CT scan.

**Figure 7:**
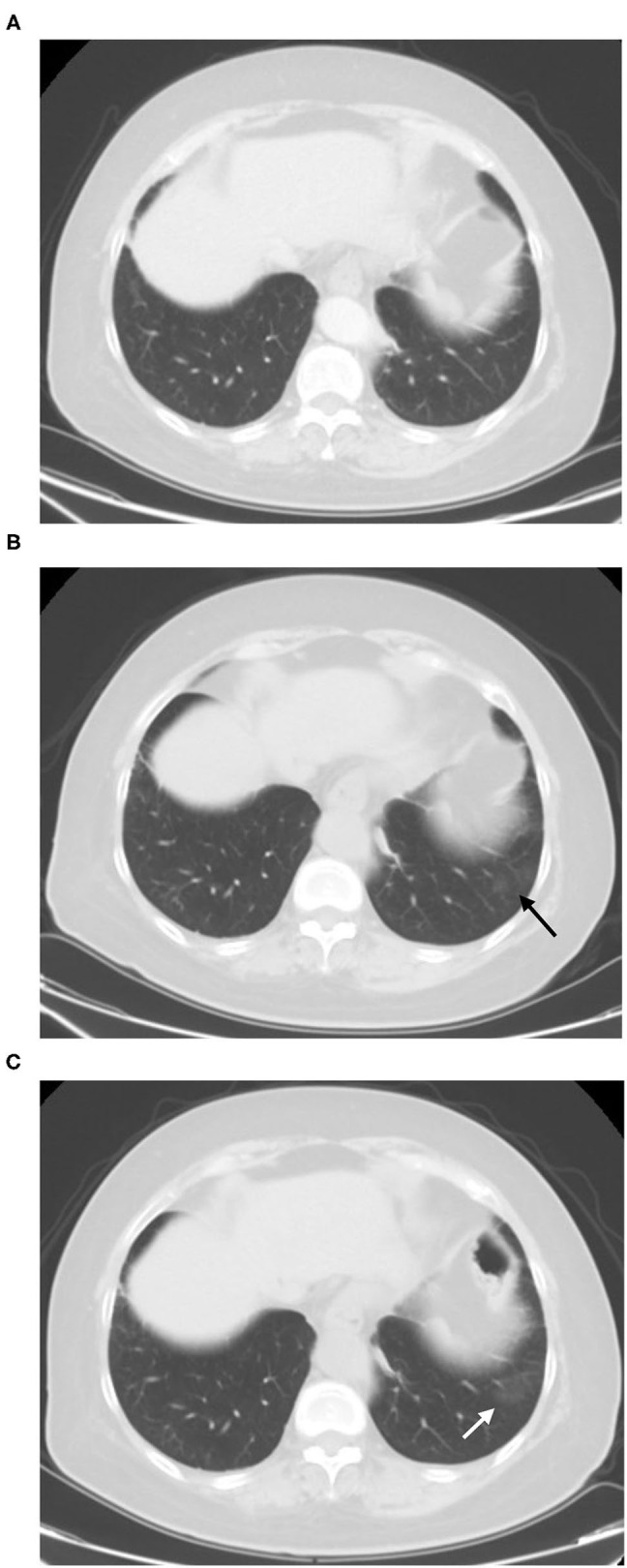
Sixty-seven-year-old female with post-target therapy of lung cancer history. Screening CT scan **(B)** showed a pure GGO lesion can be newly seen in the left lower lobe (arrow) comparison to the prior CT scan which performed at 2 months ago **(A)**. The short-term follow-up CT showed increases in the size of GGO in the lungs [**(C)**, white arrow]. The RT-PCR results of the patient was negative.

**Figure 8:**
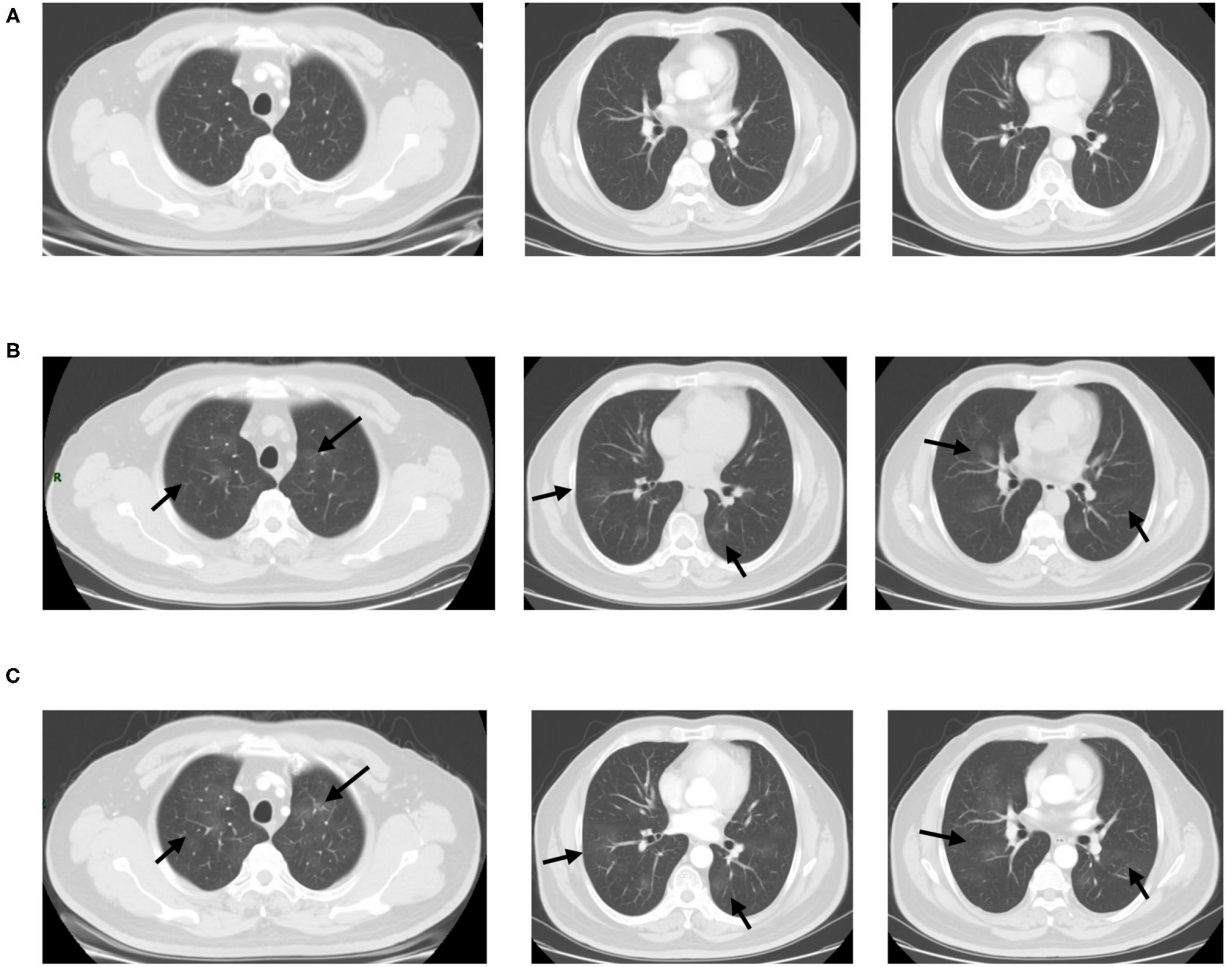
Fifty-six-year-old male with post-operation of ascending colon carcinoma history. Comparison with the prior CT which performed 1 month ago **(A)**, screening CT scans of the chest **(B)** show bilateral multiple GGOs without vascular enlargement (arrows). Most of lesions were identified in the centrally distributed. The short-term follow-up CT scan **(C)** showed unchanged (arrows). The RT-PCR results of the patient was negative.

Immediate RT-PCR was strongly recommended by the clinician for the patient, and the final test result was negative (Figure 7). Thirteen patients (26.0%, 13/50) had no change (median: 19 days, range: 6–42 days). Among these, five patients (18.5%, 5/27) showed cluster nodular lesion, three (11.1%, 3/27) showed patchy shadow, most likely due to the inflammatory, three (11.1%, 3/27) showed consolidation, most likely due to the inflammatory or atelectasis, one (3.7%, 1/27) showed sub-pleural interstitial inflammation change, and one (3.7%, 1/27) manifested multiple GGO on bilateral lung parenchyma (Figure 8). Thirteen patients (26.0%, 13/50) had resolved or partly resolved (median: 19 days, range: 6–53 days).

Among the 23 patients who had no further follow up CT scan, nine patients (39.1%, 9/23) showed patchy shadow on sub-pleural due to atelectasis or hypostatic pneumonia, seven patients (30.4%, 7/23) manifested consolidation due to chronic inflammation or atelectasis, four patients (17.4%, 4/23) manifested cluster of nodular lesion, most likely due to inflammatory, one (4.3%, 1/23) manifested focal GGO, most likely due to Atypical adenomatous hyperplasia (AAH) of lung, one (4.3%, 1/23) manifested segmental GGO, most likely due to inflammatory or AAH, and another one (4.3%, 1/23) manifested diffuse small airway lesions or respiratory bronchiolitis (All the COVID-19 screening CT finding were showed in Table 2).

**Table 2:** Morphological feature of screening CT and the results of follow-up CT scan.

	**Patients had further CT scan (*****n*** **=** **27)**			**Patients had no further CT scan (*n* = 23)**
	**Unchanged (*n* = 13)**	**Resolve or partly resolve (*n* = 13)**	**Increase in size (*n* = 1)**	
Cluster nodular lesion	5	7	1	4
GGO	1	0	0	2
Interstitial inflammation	1	0	0	0
Consolidation	3	1	0	7
Sub-plerual patchy shadow	3	5	0	9
Respiratory bronchiolitis	0	0	0	1

## Discussion

At the time of writing, although COVID-19 is still spreading rapidly overseas, all COVID-19 patients in Wuhan, where the first case was reported in the city in December 2019, had been discharged, and hospitalized COVID-19 cases had dropped to zero on April 26, 2020. A new issue is that asymptomatic patients may add a new and worrying dimension to the spread of the pandemic. Besides Wuhan city, the medical institutions of all other Chinese cities have now switched back to their normal work status to serve the medical needs of the public. Because SARS-Cov-2 has proved to have the ability for efficient human-to human transmission, hospitals are not only gathering places for suspected COVID-19 patients but also remain high-risk areas for infection. From February 1, all patients, except emergencies, were denied access to our hospital. All the patients should make an appointment by telephone, internet, or hospital appointment system. This minimized the risks to both patients and the healthcare team while reducing the utilization of unnecessary resources. All elective surgical and other anti-tumor treatments were postponed during the pandemic. In addition, postponing these services also minimized potential exposure of the COVID-19 to unsuspecting healthcare providers and patients (15). The National Cancer Center of China, also known as the Cancer Hospital, is the largest cancer-specialist hospital and research institution in Asia. Nearly 1.3 million outpatients visit it, and nine thousand inpatients receive various anti-tumor-treatment. Therefore, to minimize the effect of the epidemic on cancer patients, providing recommendations of scientific and reasonable treatment and preventive measures for cancer patients in the global epidemic scenario is an urgent requirement. Given the occurrence of recurrent waves and outbreaks of COVID-19, it is of paramount importance to resume medical services in specialized hospitals in the long run while controlling the epidemic. Nowadays, patients were required to take the nucleic acid testing before receiving treatments or examinations in all specialized hospitals in China. However, this is not practical to be included as a clinical routine procedure. Accordingly, our experiences provided a feasible pathway for the specialized hospitals to deal with the potential threat of COVID-19 in the post-COVID 19 period. We recommend those who are suspect of COVID-19 from CT images to be referred to take the nucleic acid testing or other investigations. In this context, CT scans could serve as a timely alarm for further testing and intense follow-up so that we could provide the right care, to the right patients, at the right time.

When we decided to re-admit cancer patients, the most important consideration was identification of suspected cases of COVID-19 including patients and the accompanying persons at the earliest possible stage.

Although SARS-CoV-2 nucleic acid testing remains the golden standard for the diagnosis of COVID-19, due to qualification of the specimen sampling from nasopharyngeal swab and the detection kit, the positive rate of SARS-CoV-2 nucleic acid testing is only around 30–50% (16). High-resolution CT is the optimum choice to detect possible pulmonary opacities in COVID-19 suspicious patient. At present, low dose CT is not recommended for the screening of COVID-19 due to the low imaging quality and possibility of false negative GGO detection.

In order to detect suspected viral pneumonia even COVID-19 on CT imaging, we have set up an effective mechanism of the supervisory radiologist reading system. The SR reads the initial CT images immediately after completion of the scan for each patient to determined suspected pneumonia CT characteristics. If the SR is unable to determine the pneumonia CT diagnosis, our procedure is to seek consultation from the PR. Fortunately, all the 50 cases we reported were false positive results which could be diagnosed by our thoracic-experience radiologists. To date, we have had no confirmed cases of COVID-19.

CT imaging characteristics are non-specific (14, 17). Although definitive diagnosis cannot be made on the basis of imaging features alone, especially in the COVID-19 low incidence area, in our cohort, the pneumonia lesions were predominantly peripheral and subpleural in 29 (58.0%) patients, much less than other reports (16, 18–24). GGO and consolidation were manifested only in 14 cases, much lower than the typical COVID-19 CT finding on previously reports (16, 18–23). On the contrary, sub-pleura small patchy or strip-like lesions most likely due to fibrosis or hypostatic pneumonia and cluster of nodular lesions were two main signs of suspected cases on CT images (34, 68.0%). Therefore, we could distinguish these cases as the other type of pneumonia, like other viral pneumonia, bacterial pneumonia, mycoplasma pneumonia, and many non-infectious diseases preliminarily from the COVID-19 cases. Machine-learning based methods are emerging rapidly in recent years focusing on COVID detection or disease diagnosis (24, 25). These methods may be helpful to differentiate COVID-19 from other pulmonary infection and/or non-infectious diseases (26, 27). However, our experience would provide a practical tool for health providers where machine learning is not accessible, especially in hospitals with limited medical resources.

Some patients with COVID-19 who do not receive effective therapy will demonstrate deterioration and changes will be relatively rapid. The lesions on CT will become more diffuse distributed in a truly short term (28, 29). Therefore, serial CT imaging of patients could help to diagnosis, or continuously monitor disease changes. In our cohort, 13 patients (26.0%) had no change, while 13 patients (26.0%) had resolved or partly resolved. These cases were subsequently excluded by the diagnosis of COVID-19 because of persistent existence pulmonary lesions or resolved in a short-period. Only one patient had increased in size (interval time: 8 days) compared with the further CT scan and the final RT-PCR test result was negative.

## Limitations

The study has several limitations. First, this was a single-center study, and a multicenter study and/or including more cases, especially more confirmed COVID-19 cases, are needed.

Second, we did not take throat swab samples and the blood routine examination of WBC, Neutrophil, and Lymphocyte are more suitable for finding cancer patients with asymptomatic suspected COVID-19 infection. Third, 23 (46.0%) patients had no further CT examination to evaluate the change of the CT feature. Therefore, long-term radiological follow-up is needed to confirm our findings.

## Conclusion

In conclusion, CT may be useful as a screening tool for COVID-19 based on imaging features. Confirmed diagnosis should, however, ultimately be based on the etiology. The cancer patients in low-incidence areas should continue treatment after careful screening before admission.

## Data Availability Statement

The raw data supporting the conclusions of this article will be made available by the authors, without undue reservation.

## Author Contributions

WT: conceptualization, data curation, formal analysis, investigation, methodology, writing-original draft, and writing-review and editing. NW and J-WW: conceptualization, formal analysis, investigation, methodology, supervision, funding acquisition, project administration, writing-original draft, and writing-review and editing. XZ, S-JZ, LL, and YH: formal analysis, investigation, and writing-review and editing. All authors contributed to the article and approved the submitted version.

## Funding

This study was supported by the National Key Research and Development Program of China (No. 2017YFC1308700), National Natural Sciences Foundation of China (No. 81971616), Beijing Nova Program (Z202200006820070), and Chinese Academy of Medical Sciences Initiative for Innovative Medicine (No. 2017-I2M-1-005).

## Conflict of Interest

The authors declare that the research was conducted in the absence of any commercial or financial relationships that could be construed as a potential conflict of interest.

## Publisher's Note

All claims expressed in this article are solely those of the authors and do not necessarily represent those of their affiliated organizations, or those of the publisher, the editors and the reviewers. Any product that may be evaluated in this article, or claim that may be made by its manufacturer, is not guaranteed or endorsed by the publisher.
